# DGPRI, a new liver fibrosis assessment index, predicts recurrence of AFP-negative hepatocellular carcinoma after hepatic resection: a single-center retrospective study

**DOI:** 10.1038/s41598-024-61615-0

**Published:** 2024-05-10

**Authors:** Bolun Zhang, Junshuai Xue, Bowen Xu, Jianping Chang, Xin Li, Zhen Huang, Hong Zhao, Jianqiang Cai

**Affiliations:** Department of Hepatobiliary Surgery, National Cancer Center/National Clinical Research Center for Cancer/Cancer Hospital, Chinese Academy of Medical Sciences and Peking Union Medical College, 17 Panjiayuan Nanli, Chaoyang District, Beijing, 100021 China

**Keywords:** Alpha-fetoprotein-negative hepatocellular carcinoma, Liver fibrosis, Postoperative recurrence, Gamma-glutamyl transpeptidase-to-platelet ratio, Direct bilirubin, Cancer, Oncology, Risk factors

## Abstract

Although patients with alpha-fetoprotein-negative hepatocellular carcinoma (AFPNHCC) have a favorable prognosis, a high risk of postoperative recurrence remains. We developed and validated a novel liver fibrosis assessment index, the direct bilirubin-gamma-glutamyl transpeptidase-to-platelet ratio (DGPRI). DGPRI was calculated for each of the 378 patients with AFPNHCC who underwent hepatic resection. The patients were divided into high- and low-score groups using the optimal cutoff value. The Lasso-Cox method was used to identify the characteristics of postoperative recurrence, followed by multivariate Cox regression analysis to determine the independent risk factors associated with recurrence. A nomogram model incorporating the DGPRI was developed and validated. High DGPRI was identified as an independent risk factor (hazard ratio = 2.086) for postoperative recurrence in patients with AFPNHCC. DGPRI exhibited better predictive ability for recurrence 1–5 years after surgery than direct bilirubin and the gamma-glutamyl transpeptidase-to-platelet ratio. The DGPRI-nomogram model demonstrated good predictive ability, with a C-index of 0.674 (95% CI 0.621–0.727). The calibration curves and clinical decision analysis demonstrated its clinical utility. The DGPRI nomogram model performed better than the TNM and BCLC staging systems for predicting recurrence-free survival. DGPRI is a novel and effective predictor of postoperative recurrence in patients with AFPNHCC and provides a superior assessment of preoperative liver fibrosis.

## Introduction

Liver cancer, the sixth most common tumor and the third leading cause of cancer-related deaths, poses a significant threat to human health worldwide^1^. Among primary liver cancers, hepatocellular carcinoma (HCC) is the predominant pathological type, accounting for 75–90% of cases^2–4^. Surgical resection is a vital curative approach for patients with HCC; however, the high recurrence rate within 5 years post-surgery poses significant management challenges. Abdominal ultrasonography and alpha-fetoprotein (AFP) measurement are the standard methods for diagnosing and monitoring HCC^5^. However, up to 30–40% of patients with HCC exhibit low AFP levels; this condition is defined as alpha-fetoprotein-negative hepatocellular carcinoma (AFPNHCC) when AFP levels are equal to or less than 20 ng/ml^6^. This situation significantly undermines the clinical utility of AFP. Although various studies have explored alternative tumor markers^7,8^, satisfactory indicators for diagnosis and postsurgical monitoring in this patient group are lacking. Therefore, further investigation into novel and readily accessible clinical indicators is crucial.

The occurrence and development of various types of HCC are closely linked^9^. In 1977, Japanese researchers identified a significant association between hepatitis B, cirrhosis, and HCC^10^. Consequently, liver inflammation and fibrosis play crucial roles in the diagnosis and prognosis of HCC. The gamma-glutamyl transpeptidase (GGT)-to-platelet (PLT) ratio (GPR), introduced by Lemoine et al*.* is a novel predictor of liver fibrosis and cirrhosis^11^. Numerous studies have demonstrated the utility of GPR for predicting liver fibrosis and cirrhosis^12–14^ and effective diagnosis and prognosis of HCC^15,16^. Compared with the aspartate aminotransferase platelet ratio index (APRI) and fibrosis 4 score (FIB4), GPR exhibited superior diagnostic ability for liver fibrosis in a Chinese population^17^. A meta-analysis further confirmed the high sensitivity, accuracy, and prognostic value of GPR in HCC^18^. Huang et al*.* found that GPR could be employed for diagnosing AFPNHCC^19^ and was associated with poor tumor differentiation, suggesting its potential as a valuable predictor of postoperative recurrence in these patients.

**Figure 1:**
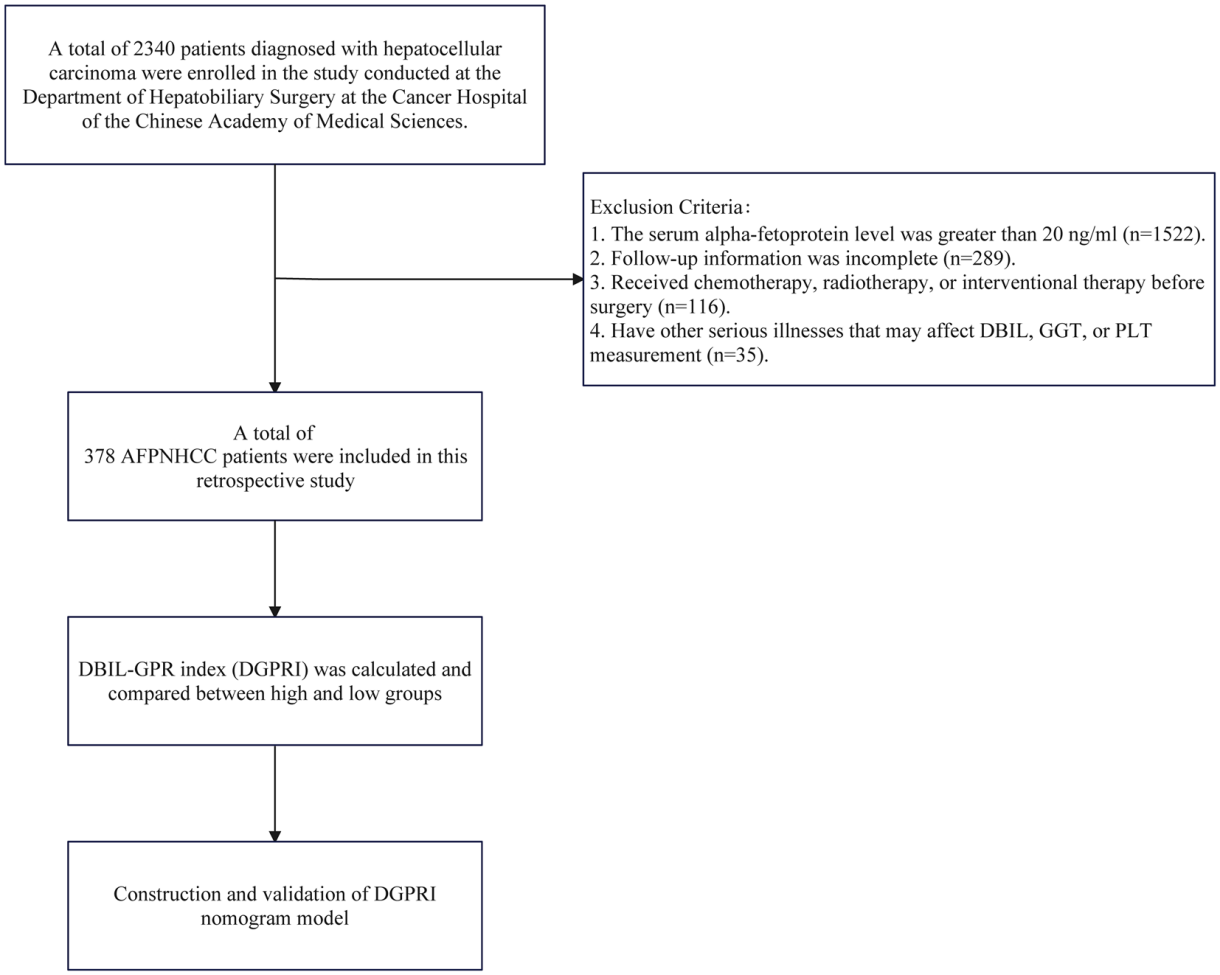
Flow chart of this retrospective study. *AFPNHCC* alpha-fetoprotein-negative hepatocellular carcinoma, *DBIL* direct bilirubin, *GGT* gamma-glutamyl transpeptidase, *PLT* platelet.

Direct bilirubin (DBIL), or conjugated bilirubin, is generated through the conversion and combination of unconjugated bilirubin and glucuronic acid in liver cells. Elevated direct bilirubin levels are associated with impaired bile excretion and poor liver function. Furthermore, as cirrhosis progresses, there is a consistent upward trend in the levels of direct bilirubin^20^, suggesting a close correlation between this marker and the degree of liver fibrosis. In this study, we developed a novel liver fibrosis-related index, the DBIL-GPR index (DGPRI), to assess its ability to predict the postoperative recurrence of HCC. We evaluated the correlation between DGPRI and the grade of liver fibrosis according to postoperative pathology, and the results demonstrated that DGPRI could reflect changes in liver fibrosis. We constructed a nomogram based on clinical features, intraoperative conditions, and pathological characteristics of patients with AFPNHCC who underwent resection to predict postoperative recurrence. To evaluate the predictive efficacy of this nomogram, we compared it with the commonly used TNM and BCLC staging systems. A flowchart of this retrospective study is shown in Fig. 1.

## Results

### Baseline information on the study cohort

A total of 378 patients with HCC and an AFP level less than or equal to 20 ng/ml, who met the inclusion and exclusion criteria, were included in the study. Among these patients, 87.8% were males and 70.6% had a history of hepatitis B virus infection. The median follow-up period was 61 months, ranging from 1 to 110 months. The recurrence-free survival (RFS) rates at the first, second, and fifth years after surgery were 92.3%, 83%, and 60.5%, respectively. Using a random number method, the patients were divided into a training set (n = 264) and a validation set (n = 114) in a ratio of 7:3. The baseline data of all the patients, including their basic profile, surgical condition, pathology, and hematological examination, are presented in Table 1, for comparison of clinicopathological differences between the two groups. The results indicated a balanced distribution of these factors between the groups, with no significant differences (p > 0.05).

**Table 1:** Comparison of baseline information of patients in the training set and validation set.

Characteristics	All patients N = 378	Training set N = 264	Validation set N = 114	p
Sex, n (%)				0.368
Male	332 (87.8%)	235 (89.0%)	97 (85.1%)	
Female	46 (12.2%)	29 (11.0%)	17 (14.9%)	
Age, n (%)				0.720
< 60	229 (60.6%)	162 (61.4%)	67 (58.8%)	
≥ 60	149 (39.4%)	102 (38.6%)	47 (41.2%)	
Family history of malignant tumors, n (%)				0.535
No	288 (76.2%)	204 (77.3%)	84 (73.7%)	
Yes	90 (23.8%)	60 (22.7%)	30 (26.3%)	
Hypertension, n (%)				0.683
No	256 (67.7%)	181 (68.6%)	75 (65.8%)	
Yes	122 (32.3%)	83 (31.4%)	39 (34.2%)	
Diabetes, n (%)				0.383
No	310 (82.0%)	220 (83.3%)	90 (78.9%)	
Yes	68 (18.0%)	44 (16.7%)	24 (21.1%)	
Smoking, n (%)				0.473
No	190 (50.3%)	129 (48.9%)	61 (53.5%)	
Yes	188 (49.7%)	135 (51.1%)	53 (46.5%)	
HBV infection, n (%)				0.618
No	111 (29.4%)	75 (28.4%)	36 (31.6%)	
Yes	267 (70.6%)	189 (71.6%)	78 (68.4%)	
HCV infection, n (%)				0.951
No	347 (91.8%)	243 (92.0%)	104 (91.2%)	
Yes	31 (8.20%)	21 (7.95%)	10 (8.77%)	
BMI, n (%)				0.611
< 28	322 (85.2%)	227 (86.0%)	95 (83.3%)	
≥ 28	56 (14.8%)	37 (14.0%)	19 (16.7%)	
Operation duration, n (%)				0.261
≤ 180	184 (48.7%)	123 (46.6%)	61 (53.5%)	
> 180	194 (51.3%)	141 (53.4%)	53 (46.5%)	
Intraoperative blood loss, n (%)				0.506
≤ 200	251 (66.4%)	172 (65.2%)	79 (69.3%)	
> 200	127 (33.6%)	92 (34.8%)	35 (30.7%)	
Intraoperative blood transfusion, n (%)				0.532
No	341 (90.2%)	236 (89.4%)	105 (92.1%)	
Yes	37 (9.79%)	28 (10.6%)	9 (7.89%)	
Number of tumors, n (%)				0.981
Single	350 (92.6%)	245 (92.8%)	105 (92.1%)	
Multiple	28 (7.41%)	19 (7.20%)	9 (7.89%)	
Edmondson Steiner grade, n (%)				0.242
I-II	310 (82.0%)	212 (80.3%)	98 (86.0%)	
III-IV	68 (18.0%)	52 (19.7%)	16 (14.0%)	
Maximum diameter of the largest tumor, n (%)				0.723
< 5	289 (76.5%)	200 (75.8%)	89 (78.1%)	
≥ 5	89 (23.5%)	64 (24.2%)	25 (21.9%)	
Liver capsule invasion, n (%)				0.324
No	216 (57.1%)	146 (55.3%)	70 (61.4%)	
Yes	162 (42.9%)	118 (44.7%)	44 (38.6%)	
MVI, n (%)				0.822
No	294 (77.8%)	204 (77.3%)	90 (78.9%)	
Yes	84 (22.2%)	60 (22.7%)	24 (21.1%)	
Satellite nodule, n (%)				0.370
No	357 (94.4%)	247 (93.6%)	110 (96.5%)	
Yes	21 (5.56%)	17 (6.44%)	4 (3.51%)	
Scheuer scoring system, n (%)				0.905
S0-S1	126 (33.3%)	89 (33.7%)	37 (32.5%)	
S2-S4	252 (66.7%)	175 (66.3%)	77 (67.5%)	
AJCC-TNM staging, n (%)				0.561
I	266 (70.4%)	186 (70.5%)	80 (70.2%)	
II	91 (24.1%)	63 (23.9%)	28 (24.6%)	
III	20 (5.29%)	15 (5.68%)	5 (4.39%)	
IV	1 (0.26%)	0 (0.00%)	1 (0.88%)	
BCLC staging, n (%)				0.244
0	69 (18.3%)	42 (15.9%)	27 (23.7%)	
A	288 (76.2%)	208 (78.8%)	80 (70.2%)	
B	16 (4.23%)	11 (4.17%)	5 (4.39%)	
C	5 (1.32%)	3 (1.14%)	2 (1.75%)	
AFP, median [IQR]	4.22 [2.55;7.08]	4.08 [2.44;7.15]	4.73 [2.73;7.03]	0.454
HGB, median [IQR]	151 [142;160]	151 [143;160]	151 [138;160]	0.440
PLT, median [IQR]	164 [129;202]	167 [133;205]	158 [118;198]	0.144
ALT, median [IQR]	26.0 [20.0;40.0]	26.0 [20.0;39.2]	28.0 [20.2;40.4]	0.588
AST, median [IQR]	25.0 [20.0;33.0]	24.4 [20.0;32.0]	26.0 [21.0;34.0]	0.575
TBIL, median [IQR]	13.6 [9.90;17.2]	13.7 [9.97;17.1]	13.4 [9.90;17.6]	0.951
DBIL, median [IQR]	4.50 [3.40;5.80]	4.50 [3.50;5.80]	4.40 [3.30;5.90]	0.656
ALB, median [IQR]	44.5 [41.6;46.8]	44.2 [41.5;46.8]	44.9 [42.2;46.9]	0.159
LDH, median [IQR]	169 [147;191]	170 [146;191]	166 [149;189]	0.874
GGT, median [IQR]	40.0 [26.0;63.9]	39.4 [26.0;62.4]	41.0 [25.2;68.8]	0.529
DGPRI, n (%)				0.673
< 1.85	177 (46.8%)	126 (47.7%)	51 (44.7%)	
≥ 1.85	201 (53.2%)	138 (52.3%)	63 (55.3%)	
Adjuvant therapy, n (%)				0.549
No	299 (79.1%)	211 (79.9%)	88 (77.2%)	
Yes	79 (20.9%)	53 (20.1%)	26 (22.8%)	

*HBV* hepatitis B virus, *HCV* hepatitis C virus, *BMI* body mass index, *MVI* microvascular invasion, *AJCC-TNM* American Joint Committee on Cancer tumor–node–metastasis, *BCLC* Barcelona Clinic Liver Cancer, *AFP* alpha-fetoprotein, *HGB* hemoglobin, *PLT* platelet, *ALT* alanine aminotransferase, *AST* aspartate aminotransferase, *TBIL* total bilirubin, ALB albumin, *LDH* lactate dehydrogenase, *DBIL* Direct bilirubin, *GGT* gamma-glutamyl transpeptidase, *DGPRI* DBIL-GPR index.

### High DGPRI is an independent risk factor for postoperative recurrence in patients with AFPNHCC

**Figure 2:**
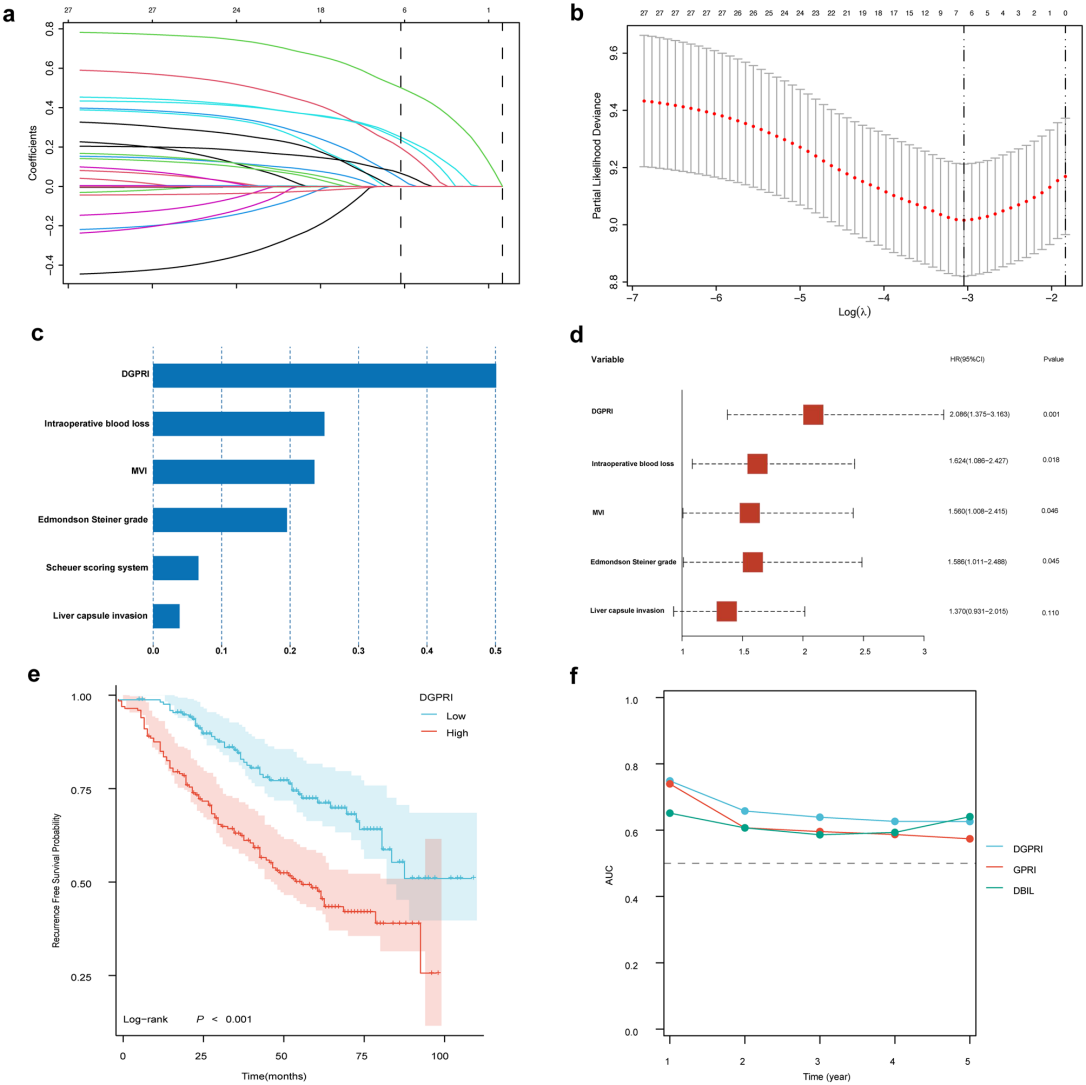
DGPRI is an independent risk factor for postoperative recurrence of AFPNHCC. (**a**–**c**) LASSO-COX feature screening process and correlation coefficient. (**d**) Multivariate Cox regression forest plot for postoperative recurrence of AFPNHCC. (**e**) Kaplan–Meier survival analysis shows that high DGPRI is associated with earlier postoperative recurrence in AFPNHCC. (**f**) Time-dependent AUC results showed that DGPRI can better predict recurrence than GPRI and DBIL at 1–5 years postoperatively. *DGPRI* DBIL-GPR index, *AFPNHCC* alpha-fetoprotein-negative hepatocellular carcinoma, *MVI* microvascular invasion, *DBIL* direct bilirubin, *GPRI* gamma-glutamyl transpeptidase to platelet ratio index, *AUC* area under curve.

Initially, all patients were classified into two groups, namely, the high DGPRI and low DGPRI groups, using a cutoff value of 1.89. In the training cohort, all baseline data were included in the least absolute shrinkage and selection operator (LASSO)-Cox regression model to identify indicators with non-zero coefficients. The DGPRI score, incidence of intraoperative hemorrhage, microvascular invasion, Edmondson-Steiner grade, hepatic peritoneal invasion grade, and hepatic fibrosis grade (Scheuer scoring system) had coefficients of 0.50, 0.25, 0.24, 0.20, 0.07, and 0.04, respectively (Fig. 2a–c). Five indicators were selected and included in the backward stepwise multivariate Cox regression analysis. High DGPRI (HR = 2.086, CI 1.375–3.163), massive intraoperative bleeding (HR = 1.624, CI 1.086–2.427), microvascular invasion (HR = 1.560, CI 1.008–2.415), and poor pathological grade (HR = 1.586, CI 1.011–2.488) were significant independent contributors to the risk of postoperative recurrence in patients with AFPNHCC (p < 0.05) (Fig. 2d). The regression model included hepatic peritoneal invasion, a crucial indicator of postoperative recurrence. Furthermore, the Kaplan–Meier curves revealed a significantly higher postoperative recurrence rate in patients with high DGPRI levels (p < 0.001; median PFS: 60 vs. 89 months) (Fig. 2e). Subsequently, we compared DGPRI with DBIL and GPR using time-dependent area under the ROC curve (AUC) analysis, which indicated that DGPRI had superior predictive ability for recurrence from 1–5 years after surgery compared to DBIL and GPR (Fig. 2f). The 1-, 2- and 5-year overall survival rates of all patients were 97.9%, 93.7% and 85.5%, respectively. Patients with high DGPRIs showed shorter overall survival (p < 0.001) (Supplementary Fig. S2).

### High DGPRI indicates worse clinicopathologic features

**Figure 3:**
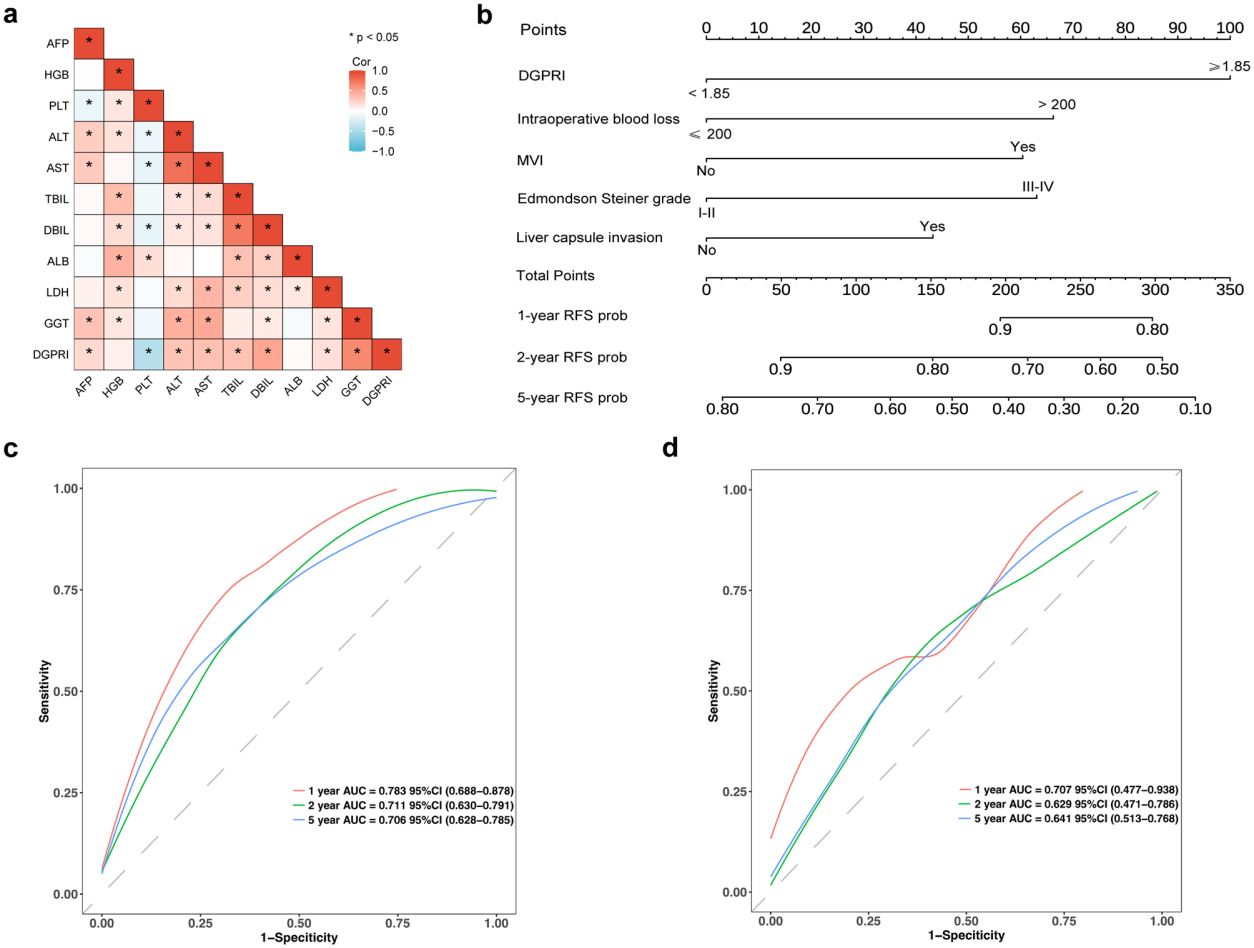
DGPRI correlational analysis and nomogram model construction. (**a**) The correlational heatmap shows that DGPRI correlates positively with AFP, ALT, AST, LDH, GGT, TBIL, and DBIL. (**b**) DGPRI-based nomogram model predicts recurrence at 1, 2, and 5 years after surgery; ROC curves of the training (**c**) and validation (**d**) sets indicate that the model can predict early postoperative recurrence in AFPNHCC. *DGPRI* DBIL-GPR index, *AFP* alpha-fetoprotein, *HGB* hemoglobin, *PLT* platelet, *ALT* alanine aminotransferase, *AST* aspartate aminotransferase, *TBIL* total bilirubin, *ALB* albumin, *LDH* lactate dehydrogenase, *DBIL* direct bilirubin, *GGT* gamma-glutamyl transpeptidase, *AFPNHCC* alpha-fetoprotein-negative hepatocellular carcinoma, *AUC* area under curve, *ROC* receiver operating characteristic.

The baseline data of all patients were compared based on the DGPRI grouping (Table 2). The results indicated no significant differences between the two groups in terms of basic general information, including age and medical history (p > 0.05). Further, there was no significant difference in the extent of surgical resection between the two groups (p = 0.890). Similarly, the number of patients receiving postoperative adjuvant therapy was similar between the two groups (p = 0.614). However, elevated DGPRIs levels were strongly associated with adverse clinicopathological characteristics, such as intraoperative bleeding exceeding 200 ml, transfusion requirement during the operation, and more severe postoperative liver fibrosis (p < 0.05). DGPRI was positively correlated with AFP, alanine aminotransferase (ALT), aspartate aminotransferase (AST), lactate dehydrogenase (LDH), GGT, total bilirubin (TBIL), and DBIL levels and negatively correlated with preoperative platelet counts according to preoperative serological tests (Fig. 3a).

**Table 2:** Comparison of baseline information of patients between high and low DGPRI groups.

Characteristics	All patients N = 378	Low DGPRI N = 177	High DGPRI N = 201	p
Sex, n (%)				0.060
Male	332 (87.8%)	149 (84.2%)	183 (91.0%)	
Female	46 (12.2%)	28 (15.8%)	18 (8.96%)	
Age, n (%)				0.186
< 60	229 (60.6%)	114 (64.4%)	115 (57.2%)	
≥ 60	149 (39.4%)	63 (35.6%)	86 (42.8%)	
Family history of malignant tumors, n (%)				0.743
No	288 (76.2%)	133 (75.1%)	155 (77.1%)	
Yes	90 (23.8%)	44 (24.9%)	46 (22.9%)	
Hypertension, n (%)				0.762
No	256 (67.7%)	118 (66.7%)	138 (68.7%)	
Yes	122 (32.3%)	59 (33.3%)	63 (31.3%)	
Diabetes, n (%)				0.244
No	310 (82.0%)	150 (84.7%)	160 (79.6%)	
Yes	68 (18.0%)	27 (15.3%)	41 (20.4%)	
Smoking, n (%)				0.178
No	190 (50.3%)	96 (54.2%)	94 (46.8%)	
Yes	188 (49.7%)	81 (45.8%)	107 (53.2%)	
HBV infection, n (%)				0.914
No	111 (29.4%)	51 (28.8%)	60 (29.9%)	
Yes	267 (70.6%)	126 (71.2%)	141 (70.1%)	
HCV infection, n (%)				0.995
No	347 (91.8%)	163 (92.1%)	184 (91.5%)	
Yes	31 (8.20%)	14 (7.91%)	17 (8.46%)	
BMI, n (%)				0.171
< 28	322 (85.2%)	156 (88.1%)	166 (82.6%)	
≥ 28	56 (14.8%)	21 (11.9%)	35 (17.4%)	
Operation duration, n (%)				0.085
≤ 180	184 (48.7%)	95 (53.7%)	89 (44.3%)	
> 180	194 (51.3%)	82 (46.3%)	112 (55.7%)	
Intraoperative blood loss, n (%)				< 0.001
≤ 200	251 (66.4%)	139 (78.5%)	112 (55.7%)	
> 200	127 (33.6%)	38 (21.5%)	89 (44.3%)	
Intraoperative blood transfusion, n (%)				0.043
No	341 (90.2%)	166 (93.8%)	175 (87.1%)	
Yes	37 (9.79%)	11 (6.21%)	26 (12.9%)	
Number of tumors, n (%)				0.304
Single	350 (92.6%)	167 (94.4%)	183 (91.0%)	
Multiple	28 (7.41%)	10 (5.65%)	18 (8.96%)	
Edmondson Steiner grade, n (%)				0.530
I-II	310 (82.0%)	148 (83.6%)	162 (80.6%)	
III-IV	68 (18.0%)	29 (16.4%)	39 (19.4%)	
Maximum diameter of the largest tumor, n (%)				1.000
< 5	289 (76.5%)	135 (76.3%)	154 (76.6%)	
≥ 5	89 (23.5%)	42 (23.7%)	47 (23.4%)	
Liver capsule invasion, n (%)				1.000
No	216 (57.1%)	101 (57.1%)	115 (57.2%)	
Yes	162 (42.9%)	76 (42.9%)	86 (42.8%)	
MVI, n (%)				0.342
No	294 (77.8%)	142 (80.2%)	152 (75.6%)	
Yes	84 (22.2%)	35 (19.8%)	49 (24.4%)	
Satellite nodule, n (%)				0.051
No	357 (94.4%)	172 (97.2%)	185 (92.0%)	
Yes	21 (5.56%)	5 (2.82%)	16 (7.96%)	
TNM staging, n (%)				0.167
I	266 (70.4%)	133 (75.1%)	133 (66.2%)	
II	91 (24.1%)	35 (19.8%)	56 (27.9%)	
III	20 (5.29%)	9 (5.08%)	11 (5.47%)	
IV	1 (0.26%)	0 (0.00%)	1 (0.50%)	
BCLC staging, n (%)				0.294
0	69 (18.3%)	31 (17.5%)	38 (18.9%)	
A	288 (76.2%)	140 (79.1%)	148 (73.6%)	
B	16 (4.23%)	4 (2.26%)	12 (5.97%)	
C	5 (1.32%)	2 (1.13%)	3 (1.49%)	
Scheuer scoring system, n (%)				< 0.001
S0-S1	126 (33.3%)	80 (45.2%)	46 (22.9%)	
S2-S4	252 (66.7%)	97 (54.8%)	155 (77.1%)	
AFP, median [IQR]	4.22 [2.55;7.08]	3.26 [2.08;6.07]	4.96 [2.90;7.84]	< 0.001
HGB, median [IQR]	151 [142;160]	151 [141;158]	152 [142;162]	0.123
PLT, median [IQR]	164 [129;202]	183 [157;221]	141 [103;177]	< 0.001
ALT, median [IQR]	26.0 [20.0;40.0]	22.2 [17.0;31.0]	30.0 [23.0;46.0]	< 0.001
AST, median [IQR]	25.0 [20.0;33.0]	22.0 [19.0;27.2]	28.0 [22.0;39.0]	< 0.001
TBIL, median [IQR]	13.6 [9.90;17.2]	11.9 [8.40;14.9]	15.4 [11.8;19.2]	< 0.001
DBIL, median [IQR]	4.50 [3.40;5.80]	3.70 [2.80;4.70]	5.40 [4.20;6.60]	< 0.001
ALB, median [IQR]	44.5 [41.6;46.8]	44.4 [41.9;46.5]	44.5 [41.4;47.4]	0.498
LDH, median [IQR]	169 [147;191]	159 [142;183]	173 [153;195]	0.001
GGT, median [IQR]	40.0 [26.0;63.9]	26.0 [20.1;37.0]	60.0 [41.0;89.0]	< 0.001
Major hepatectomy, n (%)				0.890
No	315 (83.3%)	148 (83.6%)	167 (83.1%)	
Yes	63 (16.7%)	29 (16.4%)	34 (16.9%)	
Adjuvant therapy, n (%)				0.614
No	299 (79.1%)	142 (80.2%)	157 (78.1%)	
Yes	79 (20.9%)	35 (19.8%)	44 (21.9%)	

*DGPRI* DBIL-GPR index. *HBV* hepatitis B virus, *HCV* hepatitis C virus, *BMI* body mass index, *MVI* microvascular invasion, *AJCC-TNM* American Joint Committee on Cancer tumor–node–metastasis, *BCLC* Barcelona Clinic Liver Cancer, *AFP* alpha-fetoprotein, *HGB* hemoglobin, *PLT* platelet, *ALT* alanine aminotransferase, *AST* aspartate aminotransferase, *TBIL* total bilirubin, *ALB* albumin, *LDH* lactate dehydrogenase, *DBIL* direct bilirubin, *GGT* gamma.

### Development and validation of the DGPRI-nomogram model

**Figure 4:**
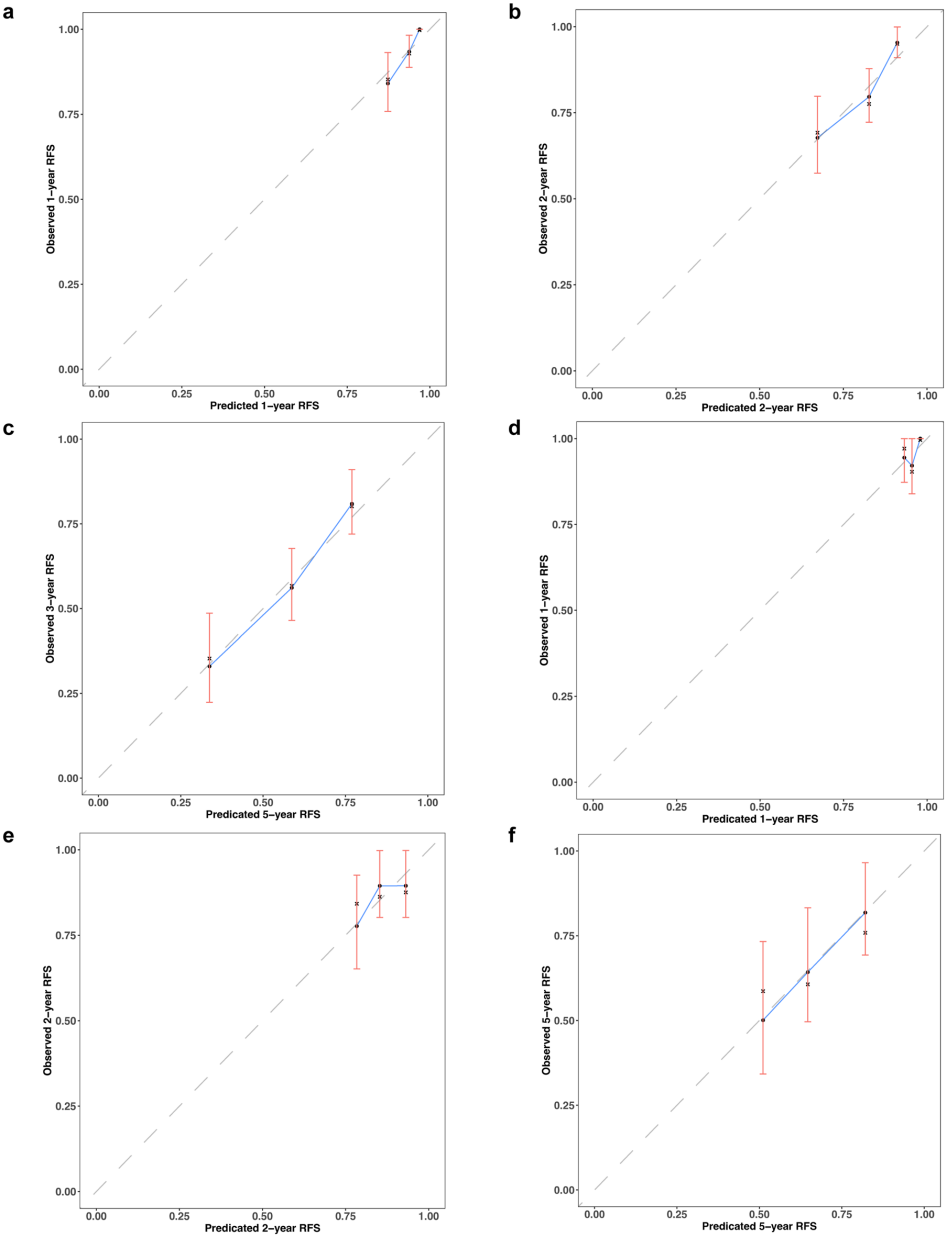
Calibration curves for the nomogram model. (**a**–**c**) Training set for 1, 2, and 5 years calibration curves versus (**d**–**f**) validation set calibration curves show that the model is consistent with the true situation.

A nomogram prediction model was constructed using DGPRI, which included the five risk factors identified from the results of multivariate Cox regression analysis (Fig. 3b). The nomogram consistency index was calculated as 0.674 (95% CI 0.621–0.727). In the training cohort, the AUC for the nomogram model at years 1, 2, and 5 was 0.783, 0.711, and 0.706, respectively (Fig. 3c). In the validation cohort, the C-index was 0.633 (95% CI 0.621–0.727), and the areas under the receiver operating characteristic (ROC) curves at 1, 2, and 5 years were 0.707, 0.629, and 0.641, respectively (Fig. 3d). The calibration curves for the model at 1, 2, and 5 years aligned with the standardized line (Fig. 4a–f). The clinical decision curves for the 1-, 2-, and 5-year survival models indicated that the nomogram exhibited a wider range of threshold probabilities than the individual metrics of DGPRI, intraoperative hemorrhage, microvascular invasion, Edmondson-Steiner grade, and hepatic perimembranous membrane invasion (see Supplementary Fig. S1a–f online). The resulting nomogram could effectively predict RFS in patients with AFPNHCC, demonstrating good agreement between the predicted and observed probabilities.

### Rationalization of the DGPRI-nomogram model

**Figure 5:**
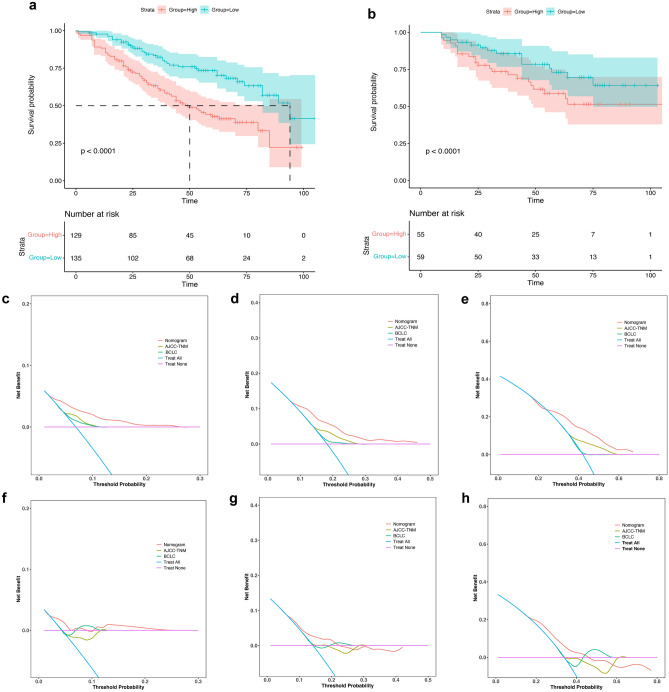
Rationalization analysis of DGPRI-nomogram model and assessment of its value for clinical application. The model can distinguish the magnitude of the risk of postoperative recurrence in the training set (**a**) and the validation set (**b**). Decision curve analysis shows that the clinical benefit of the model in the validation set is greater than that of the AJCC-TNM, BCLC staging system at 1 (**c**), 2 (**d**), and 5 (**e**) years postoperatively, and the same results are demonstrated in the validation set (**f**–**h**). DGPRI, DBIL-GPR index; *AJCC-TNM* American Joint Committee on Cancer tumor–node–metastasis, *BCLC* Barcelona Clinic Liver Cancer.

The total risk score was calculated using the nomogram prediction model, and the patients with AFPNHCC were classified into high- and low-risk groups. An optimal cutoff value of 106 points was identified for this classification. Survival curves were generated using Kaplan–Meier curves to compare RFS rates between the two groups. The results demonstrated a significant increase in postoperative recurrence in the high-risk group (P < 0.0001) (Fig. 5a). This difference was also significant in the validation cohort (Fig. 5b). When predicting tumor recurrence at 1, 2, and 5 years in patients with AFPNHCC, the decision curve analysis curves revealed that the nomogram prediction model exhibited a wider range of threshold probabilities than TNM and BCLC staging (Fig. 5c–h).

## Discussion

In this study, we modified GPR to assess hepatic fibrosis. By incorporating direct bilirubin, we developed a new hepatic fibrosis index, the DGPRI. Through Lasso-Cox screening and multivariate Cox regression, our findings revealed that DGPRI was an independent risk factor for postoperative recurrence in patients with AFP-negative HCC. Moreover, the DGPRI score correlated positively with both severe surgical trauma and postoperative pathological hepatic fibrosis grade, indicating a strong association with the extent of liver fibrosis. These results are consistent with our initial hypotheses. DGPRI showed a superior predictive capability compared to DBIL and GPR. Finally, we used the DGPRI to construct a novel nomogram model based on independent risk factors for recurrence within the cohort, showing a higher efficacy for predicting early postoperative recurrence. Notably, the ability of this model to predict RFS surpassed those of the BCLC and TNM staging systems.

HCC is the most common type of primary liver cancer and is closely associated with chronic liver disease. Patients diagnosed early and undergoing surgical resection have favorable prognosis^21^. However, the high recurrence rate after surgery poses challenges in treating and monitoring HCC^22^. AFP is the most critical biomarker for diagnosing and monitoring of HCC^23^. Elevated AFP levels are associated with increased aggressiveness and poor prognosis in patients with HCC^24^. However, approximately 30% of the patients exhibit with low AFP levels^7^. However, AFP has limited clinical utility and lacks effective biological markers for these patients' diagnosis, monitoring, and prognosis. In this study, we developed a new prognostic index, DGPRI, which was correlated with hepatic fibrosis. High DGPRI levels were associated with severe surgical injury and extensive hepatic fibrosis. Moreover, a high DGPRI could independently predict postoperative recurrence (HR = 2.086, CI 1.375–3.163), providing a strong indicator for the clinical monitoring of AFPNHCC, which is simple and easily accessible.

The DGPRI was calculated using DBIL and GPR. Direct bilirubin is a component of total bilirubin and is formed when bilirubin combines with glucuronic acid through intrahepatic glucuronosyltransferase. Elevated direct bilirubin levels are primarily caused by impaired bile excretion from biliary obstruction. In patients with cirrhosis, direct bilirubin levels increase with disease progression^20^. Elevated direct bilirubin levels are commonly observed in biliary tract malignancies or periampullary tumors and are important predictors of biliary tract tumorigenesis^25^. Xia et al. demonstrated that direct bilirubin level significantly predicts microvascular invasion in HCC. These results indicated a positive correlation between direct bilirubin levels and hepatic fibrosis, with higher levels associated with worse pathological types and poor prognosis^26^. Liver fibrosis plays a crucial role in the development of HCC^27^. The results of LASSO-Cox regression in our study showed that liver fibrosis (Scheuer scoring system) was closely associated with postoperative recurrence in patients with AFPNCC, in line with previous findings, but after multivariate correction, it was not an independent predictor of postoperative recurrence in patients with HCC, which may be because DGPRI and the hepatic fibrosis grading respond to the degree of hepatic fibrosis simultaneously, and there is a correlation between the two but the predictive efficacy of the DGPRI is higher. Recent studies suggest that the dysregulation of mitochondrial coding genes, noncoding RNAs, and nuclear alterations may contribute to the production of reactive oxygen species and inflammation, facilitating the transition from liver fibrosis to HCC^28^. Furthermore, severe hepatic fibrosis is a significant risk factor for recurrence of HCC after surgery^29^. Therefore, accurate evaluation indices for liver fibrosis are vital to precisely predict recurrence. GPR is an index commonly employed to noninvasively evaluate liver fibrosis. The GGT level was calculated as the ratio of GGT to PLT. GGT is frequently used to indicate liver function, with higher levels indicating poorer liver function. PLT, which are important substances involved in blood clotting, typically exhibit significantly elevated levels in patients with severe liver cirrhosis or fibrosis. In a study of patients with hepatitis B in West Africa, Lemoine et al*.* showed that GPR was a reliable indicator of liver fibrosis, providing a more accurate assessment of its severity than APRI and FIB-4^11^. These findings were validated in a multicenter study in China^17^. Additionally, in HCC, GPR is closely associated with poor prognosis following hepatic resection^26^. However, the underlying molecular mechanisms remain unclear. Our results demonstrate the construction of a novel hepatic fibrosis assessment index, the DGPRI, which strongly correlates with hepatic fibrosis and is consistent with the degree of fibrosis suggested by postoperative pathology. Moreover, we found significantly higher AFP levels in the high DGPRI group. Even for AFP-negative HCC, relatively high AFP levels are indicative of poor prognosis. These findings underscore the importance of regular postoperative monitoring, even in AFP-negative cases^26^. AFP is synthesized by embryonic hepatocytes, and the serum AFP level is often elevated in patients with HCC, and it is a reliable indicator for early diagnosis of HCC. Because of its high sensitivity and simple measurement, it is often used for observation and follow-up after liver cancer resection. AFP levels are widely used in determining prognoses of surgical patients. Previous studies have shown that high AFP levels are not only related to recurrence and prognosis after hepatectomy but also are significant for prognosis evaluation after liver transplantation^30–32^. However, there is a lack of effective predictors in patients with HCC and low AFP levels, which this study aimed to rectify. A previous study found that, in patients with more than five nodules, worse prognosis was seen in patients with low AFP levels. Such patients should not be considered for liver transplantation, and AFP levels should be corrected during the waiting period for liver transplantation^33^. Notably, the DGPRI was significantly positively correlated with significant intraoperative blood loss (> 200 ml). In clinical practice, the degree of liver fibrosis is closely related to massive bleeding during surgery, and severe liver cirrhosis can lead to coagulation dysfunction. At the same time, massive bleeding may lead to postoperative liver failure and perioperative infection, which are risk factors for early recurrence after hepatectomy. This suggests that a high DGPRI reflects a more severe level of cirrhosis, which leads to an increase in surgical bleeding, thereby increasing the risk of postoperative recurrence.

Usually, within 2 years after surgery, postsurgical recurrence of HCC can be categorized into early and late recurrence. Prominent factors associated with postoperative recurrence include liver fibrosis, inflammation, and liver function^34^. Xu et al. conducted a multicenter study in China to investigate the factors related to the late postoperative recurrence of HCC. Their findings indicated that sex, cirrhosis, satellite nodules, vascular invasion, and tumor size significantly contributed to the risk of late postoperative recurrence^35^. The tumor microenvironment (TME) plays a pivotal role in tumorigenesis and disease progression. Existing postoperative therapeutic strategies targeting residual tumor cells have yielded unsatisfactory outcomes. However, emerging evidence suggests that postoperative immunosuppression and specific inflammatory phenotypes within the TME are involved in the recurrence of HCC. Considering its clinical significance, the postoperative TME can serve as an adjuvant therapeutic target^36^. Inflammation-related markers can effectively predict the prognosis of patients with AFP-negative HCC after surgery^37–39^. These findings indicate a close relationship between postoperative recurrence of HCC, residual liver function, and pathological tumor characteristics. Furthermore, established high-risk factors such as previous microscopic vascular invasion (MVI) and pathological grade are significant factors for the postoperative recurrence of AFP-NHCC. Considering these factors, in addition to the novel indicators in our nomogram model, is crucial. The model demonstrated improved predictive capacity compared with the commonly used BCLC and TNM staging, possibly due to its incorporation of both tumor nature and preoperative patient baseline status (evaluated using blood markers). However, most studies have not incorporated intraoperative conditions into their predictive models. Intraoperative bleeding, blood transfusion, and hepatic portal blockade are strongly associated with the prognosis of patients due to severe ischemia–reperfusion and oxidative stress-related reactions. Our findings revealed that intraoperative bleeding exceeding 200 ml was an independent risk factor for postoperative recurrence (HR = 1.624 CI 1.086–2.427). Thus, we developed a model incorporating intraoperative, preoperative, and postoperative pathological indicators across all three dimensions, yielding more accurate and reliable predictions of postoperative recurrence in clinical practice.

This study has some limitations that warrant consideration. First, the patient's preoperative hematological indices may fluctuate, and laboratory test results collected at different time points may vary. However, the overall levels are not expected to differ significantly. Additionally, the validation set used in this study was limited to patients at our center, and external validation was lacking. Second, numerous factors affect a patient's risk of postoperative recurrence, including social relationships, social status, and family income. Acquiring precise information about these conditions is challenging in clinical settings; however, it is crucial to document them thoroughly during consultations. Furthermore, discrepancies in surgical techniques may also affect postoperative recurrence and should be emphasized in future analyses. In addition, although the nomogram developed in this study was much better than traditional HCC analysis, its predictive power was still limited. It is more clinically useful to use simple markers alone to predict prognosis, such as hemoglobin, albumin, the lymphocyte and platelet score^40^, the lymphocyte to CRP ratio^41^, the CRP-albumin-lymphocyte index^42^, and the albumin-lymphocyte-platelet-CRP index^43^; other indicators also showed good predictive ability for prognosis. Using the DGPRI to predict postoperative recurrence in patients with AFP-negative HCC may be more convenient in clinical applications. Finally, although this study preliminarily assessed differences in postoperative adjuvant therapy between high- and low-DGPRI groups, the diverse range of postoperative treatment regimens and the absence of a standard protocol hindered comparisons and were confounding factors in this study. Nonetheless, even with postoperative adjuvant therapy, patients with these high-risk recurrence factors exhibited a poor prognosis, confirming the main conclusions of this study. Therefore, a high preoperative DGPRI score can predict recurrence in AFP-NHCC patients at high risk after surgery, necessitating monitoring. In clinical practice, postoperative adjuvant therapy or neoadjuvant therapy for patients with high DGPRIs can reduce the rate of postoperative recurrence and improve overall prognosis.Further research should explore the specific underlying mechanisms and the potential benefits of preoperative neoadjuvant therapy in this patient group.

In conclusion, the DGPRI, a novel predictive index for liver fibrosis, offers improved preoperative assessment of liver fibrosis levels. It also demonstrated greater diagnostic efficacy than GPR and better predicted postoperative recurrence in patients with AFPNHCC. Moreover, it was an independent risk factor for recurrence in patients following surgery. The DGPRI nomogram model showed a high ability to predict postoperative recurrence in patients with AFPNHCC.

## Methods

### Study cohort

A total of 2340 patients diagnosed with HCC were included in this study. The patients were selected from the Department of Hepatobiliary Surgery at the Cancer Hospital of the Chinese Academy of Medical Sciences between January 2012 and November 2022. Patient data were collected from the hospital's electronic medical record system and screened based on specific inclusion and exclusion criteria. The inclusion criteria were as follows: patients who underwent hepatectomy and had confirmed postoperative pathological and histological diagnoses of hepatocellular carcinoma. Additionally, they had a preoperative serum alpha-fetoprotein level less than or equal to 20 ng/ml, lacked distant organ metastasis, and did not have concurrent malignant tumors. Patients with a serum alpha-fetoprotein level greater than 20 ng/ml, incomplete follow-up information, or prior antitumor treatments such as chemotherapy, radiotherapy, or intervention procedures were excluded. Furthermore, those with other serious conditions that could affect DBIL, GGT, or PLT measurements were excluded. These conditions included obstructive jaundice caused by biliary stones or tumors, severe infections, hematological disorders that cause platelet abnormalities, hepatitis, liver fibrosis, and autoimmune diseases. The study design adhered to the principles of the Declaration of Helsinki, and all patients provided informed consent before surgery. The research protocol was approved by the Ethics Committee of the Cancer Hospital of the Chinese Academy of Medical Sciences (Approval No. 21/198-2869).

### Acquisition of clinical data and calculation of hematologic indicators

The information of each patient included in the study, that is, the general parameters (sex, age, history of previous disease, smoking history, body mass index, peripheral blood test results (AFP, PLT, DBIL, GGT, hemoglobin, ALT, AST, TBIL, albumin, LDH) 2 weeks before the operation, intraoperative condition, and postoperative pathology, was collected. The formulae for the indicators to be calculated were as follows: GPR was calculated as (GGT (IU/L)/ upper limit of normal)/platelet count (10^9^/L) × 100. DGPRI = DBIL × GPR. Optimal group cutoff screening for operative time, intraoperative bleeding, and DGPRI was performed using the surv_cutpoint function of the Survminer package in the R software. Major hepatectomy was defined as a resection of three or more Couinaud segments^44^.

### Follow-up

Patients diagnosed with HCC underwent regular standardized postoperative follow-ups based on pathological diagnoses. The first postoperative review was performed within 1 month of surgery. Subsequent reviews were conducted every 3 months for 2 years. If there was no tumor recurrence within 2 years, the frequency of reviews was extended to every 6 months. For patients with no recurrence after 5 years, the follow-up was extended to once a year. In cases of recurrence, patients received treatment according to a standardized regimen. Follow-up assessments included obtaining a detailed medical history; conducting a physical examination; evaluating AFP levels and liver function, and performing abdominal imaging (enhanced MRI of the upper abdomen and enhanced CT of the thorax, abdomen, and pelvis). Additionally, supplemental gadoxetic acid disodium (Gd-EOB-DTPA) magnetic resonance imaging was performed to identify intrahepatic nodules that were difficult to detect. The primary endpoint of the study was tumor recurrence, and RFS was defined as the interval between the date of surgery and recurrence, death, or the last follow-up. We also recorded the postoperative adjuvant therapy of patients during the postoperative follow-up.

### Statistical analysis

The Mann–Whitney U test was used to compare differences between groups for continuous variables, while the Pearson chi-square test or Fisher’s exact test was used for subtyped variables. Statistical analyses were conducted using the R software (version 4.2.1). The optimal cutoff values for operative time, bleeding, DGPRI, and nomogram scores were determined using the Surv cut-off function of the Survminer package. A multivariable L1 LASSO-penalized Cox regression model was used to select predictive factors. Subsequently, multivariate Cox regression analysis was performed to identify independent predictors of RFS. Survival analysis was performed using Kaplan–Meier analysis with the log-rank test. Time-dependent subject operating characteristic (time-ROC) analysis was conducted to estimate the AUC at 1, 2, 3, 4, and 5 years postoperatively to assess the utility of DGPRI, GPR, and DBIL to predict RFS. Furthermore, differences in the indicators between the high- and low-DGPRI groups were compared. Correlations between variables in the dataset were analyzed using Spearman’s correlation analysis, which involved a two-by-two correlation analysis. The outcomes of this analysis were presented using heat maps for visualization. A probabilistic nomogram model for RFS was developed based on the results of multivariate Cox analysis. The nomogram’s performance was evaluated using calibration curves, ROC curves, and clinical decision analysis. The samples were categorized into high- and low-risk groups based on the model scores, and Kaplan–Meier curves were plotted to compare survival differences between the two groups. Finally, the newly constructed nomogram was compared with commonly used TNM and BCLC staging systems. Statistical significance was set at p < 0.05.

### Supplementary Information

Supplementary Figures.

## Data Availability

The datasets generated during and/or analysed during the current study are available from the corresponding author on reasonable request.
