# Supplementary material for: DGPRI, a new liver fibrosis assessment index, predicts recurrence of AFP-negative hepatocellular carcinoma after hepatic resection: a single-center retrospective study
Source: Sci Rep. 2024 May 10;14:10726. doi: 10.1038/s41598-024-61615-0 (PMC11087499; doi:10.1038/s41598-024-61615-0)
Supplement: Supplementary file 1 — Supplementary Figures. [file 41598_2024_61615_MOESM1_ESM.docx]

**DGPRI, a New Liver Fibrosis Assessment Index, Predicts Recurrence of AFP-Negative Hepatocellular Carcinoma After Hepatic Resection: A Single-Center Retrospective Study**

Bolun Zhang, Junshuai Xue, Bowen Xu, Jianping Chang, Xin Li, Zhen Huang, Hong Zhao, Jianqiang Cai*

Department of Hepatobiliary Surgery, National Cancer Center/National Clinical Research Center for Cancer/Cancer Hospital, Chinese Academy of Medical Sciences and Peking Union Medical College, Beijing, China

*Corresponding Author:

Jianqiang Cai

Department of Hepatobiliary Surgery, National Cancer Center/National Clinical Research Center for Cancer/Cancer Hospital, Chinese Academy of Medical Sciences and Peking Union Medical College

17 Panjiayuan Nanli, Chaoyang District

Beijing, 100021, China

Tel: 86-10-87787100

E-mail: caijianqiang@cicams.ac.cn


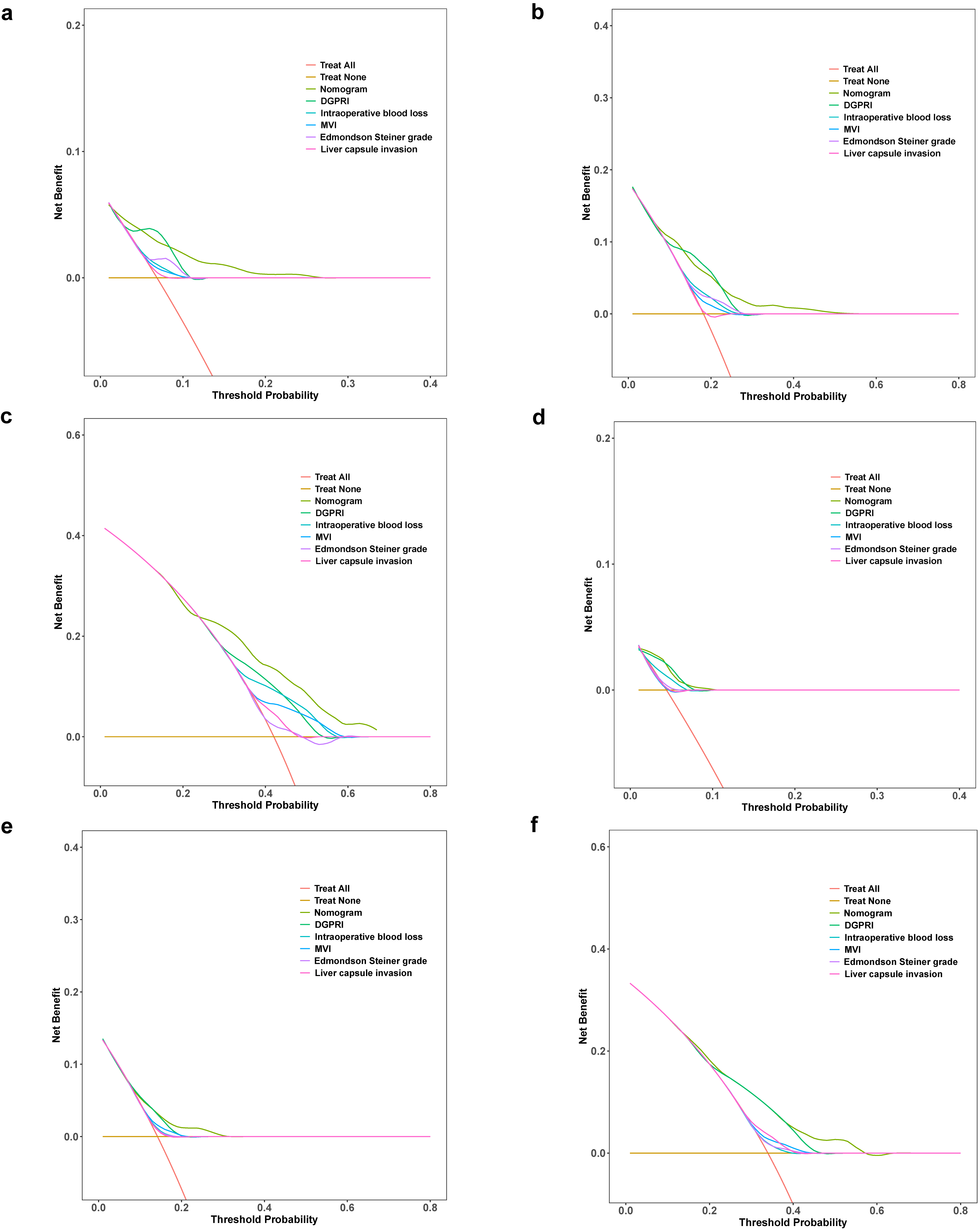


**Supplementary Figure S1** Decision analysis curves showing that in the training group, the net clinical benefit of the DGRPI-nomogram model is greater than the individual component metrics in predicting recurrence at 1 (**a**), 2 (**b**), and 5 (**c**) years after surgery. The same result is obtained in the validation set (**d-f**).


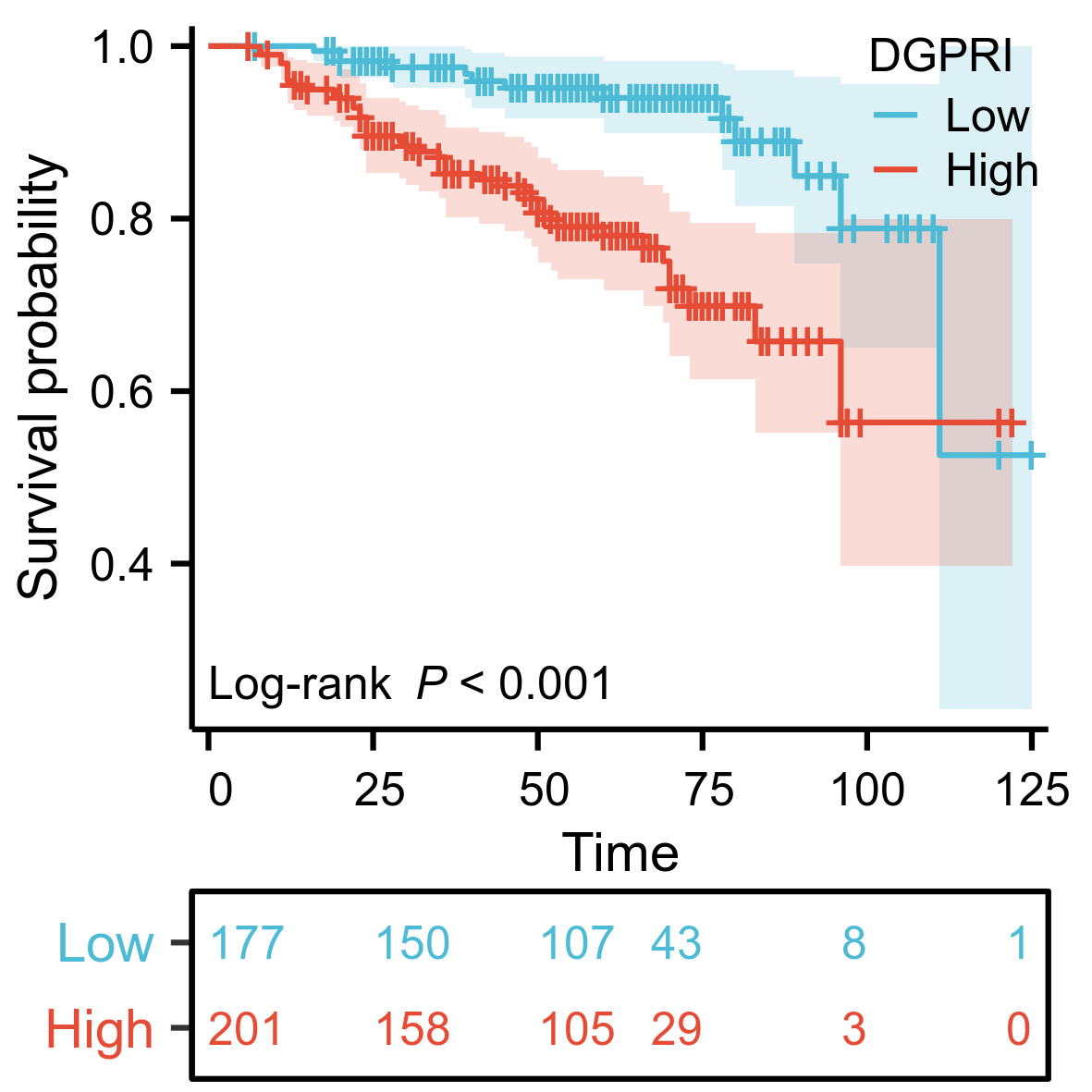


**Supplementary Figure S2** Kaplan–Meier curve showing that patients with high DGPRI had shorter overall survival
